# Detection of early decayed oranges by structured-illumination reflectance imaging coupling with texture feature classification models

**DOI:** 10.3389/fpls.2022.952942

**Published:** 2022-08-10

**Authors:** Zhonglei Cai, Wenqian Huang, Qingyan Wang, Jiangbo Li

**Affiliations:** ^1^College of Mechanical and Electrical Engineering, Shihezi University, Shihezi, China; ^2^Intelligent Equipment Research Center, Beijing Academy of Agriculture and Forestry Sciences, Beijing, China

**Keywords:** citrus, early decay detection, structured light imaging, image processing, classification models

## Abstract

Citrus fruits are susceptible to fungal infection after harvest. To reduce the economic loss, it is necessary to reject the infected citrus fruit before storage and transportation. However, the infected area in the early stage of decay is almost invisible on the fruit surface, so the detection of early decayed citrus is very challenging. In this study, a structured-illumination reflectance imaging (SIRI) system combined with a visible light-emitting diode (LED) lamp and a monochrome camera was developed to detect early fungal infection in oranges. Under sinusoidal modulation illumination with spatial frequencies of 0.05, 0.15, and 0.25 cycles mm^–1^, three-phase-shifted images with phase offsets of − 2π/3, 0, and 2π/3 were acquired for each spatial frequency. The direct component (DC) and alternating component (AC) images were then recovered by image demodulation using a three-phase-shifting approach. Compared with the DC image, the decayed area can be clearly identified in the AC image and RT image (AC/DC). The optimal spatial frequency was determined by analyzing the AC image and pixel intensity distribution. Based on the texture features extracted from DC, AC, and RT images, four kinds of classification models including partial least square discriminant analysis (PLS-DA), support vector machine (SVM), least squares-support vector machine (LS-SVM), and k-nearest neighbor (KNN) were established to detect the infected oranges, respectively. Model optimization was also performed by extracting important texture features. Compared to all models, the PLS-DA model developed based on eight texture features of RT images achieved the optimal classification accuracy of 96.4%. This study showed for the first time that the proposed SIRI system combined with appropriate texture features and classification model can realize the early detection of decayed oranges.

## Introduction

Citrus is one of the most popular fruits all over the world. Decay caused by fungi is one of the most important pathologies affecting the storage and marketing of citrus fruit. During storage or long-distance transportation, a few decayed citruses can lead to infection of the whole batch without proper precautions and controls (Folch-Fortuny et al., 2016). Due to the lack of effective detection ways, fungal infection has caused huge economic losses to the citrus industry. However, the early detection of decay is difficult because the decayed area in the initial stage is very slight with a similar appearance to the surrounding healthy tissue (Li et al., 2019). Although many grading systems have been successfully developed for commercial grading of citrus quality such as size, color, weight, etc., it remains a challenge to detect decay in the early stage of development with few visual symptoms for the citrus industry. Therefore, it is very attractive for developing an automated detection system to facilitate early decay detection and improve the profitability of the citrus industry.

In order to realize the effective and rapid detection of citrus with early decay, many techniques have been developed. Blasco et al. (2000) used near-infrared spectroscopy to detect the early decay of citrus caused by fungal infection and found that there was a spectrum difference between the healthy and decayed areas of citrus. Lorente et al. (2015a) combined near-infrared spectroscopy with intelligent learning methods to detect infected fruit and obtained good results. Their research indicated that near-infrared spectroscopy can be used to detect decay tissue. However, the infection location of decayed citrus fruit cannot be determined in advance, so this technology has limitations in practical application due to local information acquisition capability. In contrast, the imaging technique is more suitable for the detection of early decay. Blasco et al. (2007) used a machine vision system to measure citrus with different damage types and achieved a high success rate in other defects, but it was less than 60% for decay detection by (red, green, and blue) RGB imaging technique. It may be that the similar color features between the decayed and the healthy area reduce the recognition ability of the RGB imaging system. Fluorescence imaging provides another means for decay detection of citrus, based on the observation that citrus epidermal tissues can emit yellow fluorescence under excitation of ultraviolet (UV)-A light (Obenland et al., 2010). Kurita et al. (2009) designed a double image acquisition system with visible and UV LEDs for decay detection of citrus fruit. However, not all the decayed areas of citrus produce detectable fluorescence, ensuring the effectiveness of this imaging technique (Momin et al., 2012). Moreover, some citrus defects, such as peel scratch and freezing injury (Slaughter et al., 2008; Obenland et al., 2010), can produce similar fluorescence under the induction of UV-A light, which would confound the decay detection. In addition, Lorente et al. (2015b) used laser backscattering imaging to detect the early decay of citrus fruit. Statistical and physical contour modeling was used to obtain good classification results. However, the position of the decayed area needs to be matched with the laser light source and camera in order to capture a clear speckle image. Compared with the single near-infrared spectroscopy and imaging technology, hyperspectral imaging combines image and spectral information. In recent years, it has also been used to detect the early decay of citrus. Li et al. (2016) successfully detected and visualized the early decay in citrus using Vis-NIR hyperspectral imaging, with a detection accuracy of 98.6%. Li J. B. et al. (2022) proposed two-wavelength image detection of early decayed oranges by coupling spectral classification with image processing. Hyperspectral data variable selection and image principal component analysis were used to select two wavelength images for the development of a fast multispectral algorithm, and the overall classification accuracy of 96.6% was achieved. However, due to the long processing time and expensive equipment, hyperspectral imaging is mainly used for laboratory research (Wang et al., 2021). Although efforts have been made to develop hyperspectral techniques into a multispectral system for detecting decayed citrus fruit, there are no reports of multispectral imaging systems available.

In recent years, an emerging structured-illumination reflectance imaging (SIRI) technique has been developed for enhanced detection of sub-surface or near-surface slight defects in horticultural products (Lu et al., 2016). Compared with traditional uniform diffuse illumination, SIRI uses the modulated structured illumination for sample image acquisition, which can control the depth of light penetration into tissue by changing the spatial frequency of illumination, making it possible to better detect fruit surface defects. Based on the SIRI technique, a few phase-shifted images at a certain spatial frequency were obtained, and then demodulated into a direct component (DC, corresponding to uniform/diffuse illumination) and alternating component (AC, unique to structured illumination) images for processing (Lu and Lu, 2019). Different from the DC image, AC encodes depth-specific information with image contrast and resolution varying with the spatial frequency of illumination patterns. The enhanced detection ability of this technology for defects without obvious signs has been demonstrated in previous studies, such as subsurface bruising in apples (Lu and Lu, 2017a; Li et al., 2018), early decay of peaches (Sun et al., 2019), and subsurface bruising in fresh pickling cucumbers (Lu et al., 2021). However, the feasibility of this technology still needs to be verified for the detection of early decayed citrus fruit. In this study, a new SIRI system based on a visible LED lamp and a monochrome camera was developed for the detection of oranges with early decay. The corresponding classification models combined with texture features were also established. This may be the first important attempt to detect decayed citrus using SIRI technology coupled with a texture feature classification model. It is expected that the findings in this work could potentially be used to develop a low-cost and real-time SIRI system for the quality detection of fruits and other agricultural products.

The main objectives of recent work were to (1) acquire the SIRI of oranges under the sinusoidal modulation illumination with the spatial frequencies of 0.05, 0.15, and 0.25 cycles mm^–1^ using a developed SIRI system with a visible LED lamp, which was demodulated to recover DC and AC; (2) extract the texture features from DC, AC, and ratio (RT) images and optimize features by x-loading analysis to obtain the most relevant ones for the identification of decayed and healthy oranges; (3) develop feature classification models coupled with PLS-DA, SVM, LS-SVM, and KNN classifiers; and (4) evaluate the feasibility of the developed classification models and SIRI system for detecting oranges with early decay.

## Materials and methods

### Experimental samples

**FIGURE 1:**
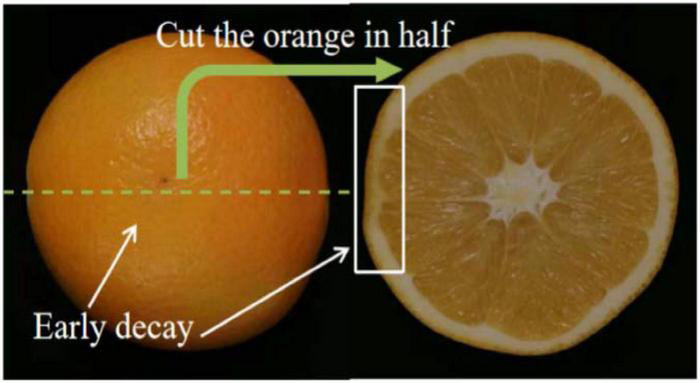
Orange sample with early decay.

Oranges were purchased from a local fruit supermarket in Beijing in December 2021. A total of 280 intact samples were prepared in this experiment. Among them, 150 samples were injected with a fungal solution of *Penicillium digitatum* and placed in the laboratory (about 25°C and 99% relative humidity) for 3 days to form early decayed samples. All samples with early decay showed no noticeable visual symptoms (e.g., severe watery and fungal spore) on the fruit surface prior to image acquisition. For the detailed preparation process of early decay samples, refer to our previous research (Li et al., 2016, 2019). Figure 1 shows photographs of a typical decayed sample (left) and the corresponding section (right). The sectional view was obtained by cutting the sample along the green line passing through the decayed area. By visually observing the intact sample, the decayed area of the sample was similar in appearance to the surrounding sound area, indicating that the identification of decayed fruit was difficult by the naked eyes and traditional RGB machine vision technology. After cutting the sample along the green line, it can be seen from the sectional view that the subcutaneous spongy tissue of the affected area had begun to decay into dark yellow. In this study, 100 healthy samples and 100 decayed samples were randomly selected as the calibration set to develop the models, whereas the remaining 80 samples (30 healthy and 50 decayed samples) were used as the test set to evaluate the performance of the models.

### The structured-illumination reflectance image acquisition

**FIGURE 2:**
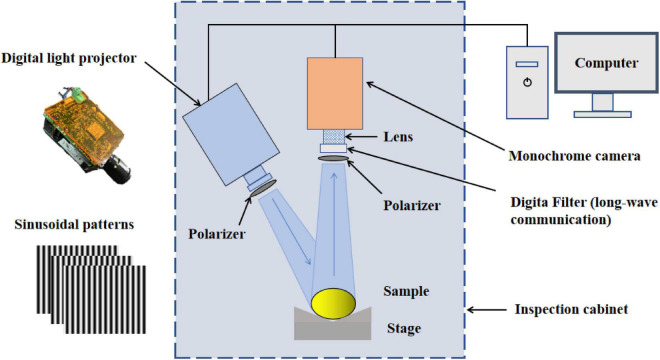
Schematic diagram of the developed structured-illumination reflectance imaging (SIRI) system.

A SIRI system based on a visible LED lamp and monochrome camera, shown in Figure 2, was developed for the acquisition of the SIRIs of all samples. The SIRI system mainly consisted of a computer (Tianyi-510S, Lenovo Inc., China), a digital light projector (DLP4500, Texas Instruments, Dallas, TX, United States) with a visible LED lamp, a monochrome camera (MV-CA050-10GM, Hangzhou Hikrobot Intelligent Technology Co., Ltd., Hangzhou, China) with an adjustable focal length lens (MVL-MF1628M-8MP, Hangzhou Hikrobot Intelligent Technology Co., Ltd., Hangzhou, China), a set of polarizers (PL-D50, RAYAN Technology Co., Ltd., Changchun, China), a long wave pass filter (GCC-300701, Daheng New Epoch Technology Inc., Beijing, China), and an adjustable stage for holding samples (600LW-WT, Shanghai Weimu Automation Equipment Co., Ltd., Shanghai, China). The camera was located above the sample and perpendicular to the sample, whereas the projector was located at the upper left of the sample and has an incident angle of 15°. The digital light projector and the camera were connected with the computer through data lines, respectively. A band-pass filter with a central wavelength of 680 nm was installed in front of the lens considering there was a significant difference between the decay and healthy tissues in this band (Li et al., 2016). In addition, two linear polarizers, attached in the front of the lens and the digital light projector, respectively, were used to suppress specular reflection from the sample surface.

In terms of the SIRI technique, the ability of light to penetrate into biological tissue is directly related to the spatial frequency of structured light; therefore, it is important to select the appropriate structured light frequency for the accurate detection of decayed oranges. Light penetration depth in the tissue decreases with the increase of spatial frequency, whereas the resolution and contrast of the acquired images show an opposite trend. The skin of oranges contains many lenticels, which can be enhanced in high-frequency structured light images and negatively affect the accurate segmentation of decayed areas, especially for slight decay. Therefore, three spatial frequencies including 0.05, 0.15, and 0.25 cycles mm^–1^ were selected on the basis of pre-experiment and finally used in this study. For structured light image acquisition, an orange was placed on the sample stage and made the decayed area (if the sample is a decayed fruit) toward the camera. Three-phase-shifted sinusoidal illumination patterns (8-bit grayscale bitmap image) with phase offsets of − 2π/3, 0, and 2π/3 shown in Figure 2, pregenerated using MATLAB software, were uploaded to the projector to illuminate the tested sample one at a time. At the same time, the camera collected structured light images of the tested sample. In this way, a total of 9 SIRIs (3 phases × 3 spatial frequencies) were acquired for each sample. Note that the three-step phase-shifting technique requires the same relative phase shift in a sinusoidal period (2π) (Schreiber and Bruning, 2007).

### Image demodulation and enhancement

#### Image demodulation

The original structured light image cannot be directly used for the recognition of decayed oranges due to the obvious light fringe in the image. Therefore, the original three-phase images collected by the SIRI system need to be demodulated to retrieve AC and DC images without any fringe. The DC image was equivalent to the image obtained under uniform illumination, whereas the AC image contained specific depth information related to the spatial frequency of the sinusoidal illumination pattern. Here, the classical three-phase-shifting approach (Carlson and Crilly, 2010) was used to retrieve AC and DC images by demodulating raw three-phase-shifted pattern images at each spatial frequency for each sample. The demodulation equations were as follows:


(1) $$AC=\frac{\sqrt{2}}{3}{\left[{\left(I_{1}-I_{2}\right)}^{2}+{\left(I_{2}-I_{3}\right)}^{2}+{\left(I_{3}-I_{1}\right)}^{2}\right]}^{\frac{1}{2}}$$

(2) $$DC=\frac{1}{3}\left(I_{1}+I_{2}+I_{3}\right)$$

where *I*_1_, *I*_2_, and *I*_3_ represent the three-phase-shifted reflectance images acquired under sinusoidal illumination with phase offsets of − 2π/3, 0, and 2π/3, respectively.

#### Image enhancement

The original DC and AC images obtained after demodulation usually have low gray value, which has a negative impact on the effective recognition of decayed areas. The RT image has been proven to be an effective image enhancement way for structured light image processing by significantly reducing image vignetting and enhancing the features of the target area of interest (Lu and Lu, 2019; Lu et al., 2021). Therefore, the RT image was also obtained by calculating the ratio of AC to the corresponding DC image. The following equation was used to calculate an RT image.


(3) $$RT=\frac{I_{AC}}{I_{DC}}$$

Where *I*_*AC*_ and *I*_*DC*_ represent the AC and DC images obtained by demodulating three-phase-shifting images, respectively.

### Image features extraction and optimization

The texture features of different images including DC, AC, and RT were extracted and analyzed. Texture features were reflected by the gray distribution of pixels and their spatial fields. Gray level co-occurrence matrix (GLCM) was one of the most widely used texture extraction methods. The main steps of texture feature extraction based on GLCM were as follows: (1) gray image acquisition; (2) gray level compression, compressing the image to eight gray levels; (3) determining the calculation parameters with horizontal direction (θ = 0°) and spacing distance (*d* = 1); (4) calculate gray co-occurrence matrix; and (5) calculate the eigenvalues. Fourteen GLCM statistic features proposed by Haralick et al. (1973) were used in this study. These parameters mainly involved angular second moment, contrast, correlation, the sum of squares: variance, inverse difference moment, sum average, sum variance, sum entropy, entropy, difference variance, difference entropy, information measures of correlation, and the maximal correlation coefficient. All parameters are listed in Table 1.

**TABLE 1:** The extracted 14 texture features.

Number	Features	Equation
1	Angular Second Moment	$f_{1}=\sum_{i}\sum_{j}{\left\{p\left(i,j\right)\right\}}^{2}$
2	Contrast	$f_{2}=\sum_{n=0}^{N_{g-1}}n^{2}\;\{\sum_{i=1}^{N_{g}}\sum_{j=1}^{N_{g}}p\left(i,j\right)\};\left|i-j\right|=n$
3	Correlation	$f_{3}=\frac{\sum_{i}\sum_{j}\left(i,j\right)p\left(i,j\right)-\mu_{x}\mu_{y}}{\sigma_{x}\sigma_{y}}$
4	Sum of Squares: Variance	$f_{4}=\sum_{i}\sum_{j}{\left(i-\mu \right)}^{2}p\left(i,j\right)$
5	Inverse Difference Moment	$f_{5}=\sum_{i}\sum_{j}\frac{1}{1+{\left(i-j\right)}^{2}}p\left(i,j\right)$
6	Sum Average	$f_{6}=\sum_{i=2}^{2N_{g}}ip_{x+y}\left(i\right)$
7	Sum Variance	$f_{7}=\sum_{i=2}^{2N_{g}}{\left(i-f_{8}\right)}^{2}p_{x+y}\left(i\right)$
8	Sum Entropy	$f_{8}=-\sum_{i=2}^{2N_{g}}p_{x+y}\left(i\right)\;\log\left\{p_{x+y}\left(i\right)\right\}$
9	Entropy	$f_{9}=-\sum_{i}\sum_{j}p\left(i,j\right)\;\log\left(p\left(i,j\right)\right)$
10	Difference Variance	*f*_10_ = *variance of p*_*x*−*y*_
11	Difference Entropy	$f_{11}=-\sum_{i=0}^{N_{g-1}}p_{x-y}\left(i\right)\;\log\left\{p_{x-y}\left(i\right)\right\}$
12–13	Information Measures of Correlation	$f_{12}=\frac{HXY-HXY1}{max\left\{HX,HY\right\}}$ *f*_13_ = (1 − *exp* [−2.0 (*HXY*2 − *HXY*)])^1/2^
14	Maximal Correlation Coefficient	*f*_14_ = (*Second largest eigenvalue of Q*)^1/2^

*p*(*i*, *j*) = (*i*, *j*)*th* entry in a normalized gray-tone spatial-dependence matrix;		
N_*g*_ = Number of distinct gray levels in the quantized image;		
$p_{x}\;(i)=\sum_{j=1}^{N_{g}}p\left(i,j\right);p_{y}\;(j)=\sum_{i=1}^{N_{g}}p\left(i,j\right);$		
$p_{x+y}\;(k)=\sum_{i=1}^{N_{g}}\sum_{j=1}^{N_{g}}p\left(i,j\right),i+j=k,k=2,3,\ldots ,2N_{g};$		
$p_{x-y}\;(k)=\sum_{i=1}^{N_{g}}\sum_{j=1}^{N_{g}}p\left(i,j\right),\left|i-j\right|=k,k=2,3,\ldots ,N_{g}-1;$		
$HXY=-\sum_{i}\sum_{j}p\left(i,j\right)\;\log\left(p\left(i,j\right)\right);HXY1=-\sum_{i}\sum_{j}p\left(i,j\right)\;\log\left(p_{x}\;(i)\;p_{y}\;(j)\right);$		
$HXY2=-\sum_{i}\sum_{j}p_{x}\left(i\right)p_{y}\left(j\right)\;\log\left(p_{x}\left(i\right)p_{y}\left(j\right)\right);Q\left(i,j\right)=\sum_{k}\frac{p\left(i,k\right)p\left(j,k\right)}{p_{x}\left(i\right)p_{y}\left(k\right)}.$		

Feature optimization is important for establishing a robust and fast machine learning model. The useless features not only affect the training speed of the model but also negatively affect the identification performance of the model. In this study, x-loading weight analysis based on PLS-DA (Liu et al., 2008) was used to select effective features from all fourteen textural features. Those features that have large absolute values of loading weight and locate at the peak or valley of the loading curve were selected as important features for the identification of decayed oranges.

### Classification models

Four kinds of classifiers including the partial least square discriminant analysis (PLS-DA), support vector machine (SVM), least squares-support vector machine (LS-SVM), and k-nearest neighbor (KNN) were used to build classification models. PLS-DA was often used for the classification and discrimination of multivariable data. It is a linear classification method combining the properties of partial least squares regression with the discrimination power of a classification technique (Haaland and Thomas, 1988; Wold et al., 2001). This method established models between multivariate data (texture features here) and a vector coding different classes (decayed and healthy oranges here). As shown in Table 2, the two types of samples, including healthy and decayed samples, were assigned as 1 and 0, respectively. The predicted value of the PLS-DA model is a real number, not a dummy integer. Therefore, it is necessary to set a cutoff value to assist in determining the classification results of the model. The cut-off value was set as 0.5. Additionally, in this study, the PLS-DA model with leave-one-out CV was applied to prevent overfitting of the calibration model (Li L. J. et al., 2022). The optimal number of latent variables (LVs) was determined by the lowest value of the predicted residual error sum of squares. SVM is a classifier with the largest interval defined in the feature space. It gives the largest minimum distance to the training data set (Fan et al., 2020). The radial basis function (RBF) was chosen as the kernel function and determined the optimal parameters (penalty coefficient C and density distribution coefficient g) through 10-fold cross-validation and mesh optimization. LS-SVM is an improved form of SVM, which can deal with linear and non-linear multivariate analysis. Compared with SVM, LS-SVM can simplify the complexity of calculation and improve the operation speed, so as to improve the analysis ability of high-dimensional data. RBF was also used as the kernel function of the LS-SVM model. The main parameters γ and σ^2^ of the LS-SVM model were determined by 10-fold cross-validation and mesh optimization. KNN is a statistical method of pattern recognition. It classifies by measuring the distance between different eigenvalues and uses the cross-validation method to select the optimal *K* value. In this study, all the classification models were developed using MATLAB R, 2020b (The MathWorks, Inc., Natick, MA, United States).

**TABLE 2:** Sample type and classification assignment.

Sample class	No. of samples	Calibration set	Test set	Assigned class
Healthy	130	100	30	1
Decayed	150	100	50	0

### Identification of decayed oranges

**FIGURE 3:**
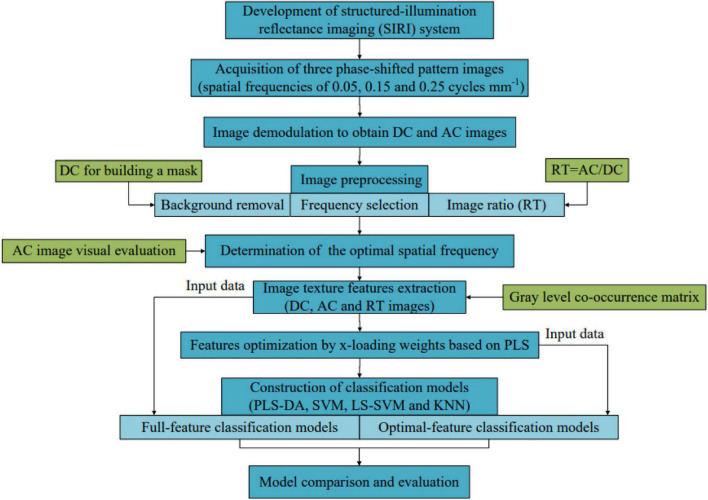
Flowchart for detection of the decayed oranges.

Figure 3 shows the flowchart for the identification of decayed oranges. First, the SIRI system based on a visible LED lamp and a monochrome camera was developed and used to obtain three-phase-shifted images of each sample at three spatial frequencies of 0.05, 0.15, and 0.25 cycles mm^–1^. The classical three-phase-shifting scheme was further used to demodulate the structured light image to obtain DC and AC images. Then, the DC image was used to produce a binary mask image by setting a threshold, and the mask image was used to remove the background of DC and AC images. Based on DC and AC images after background removal, the RT image was also obtained. The best spatial frequency was determined by evaluating AC images and the pixel intensity distribution curves corresponding to the solid lines in AC images. Subsequently, fourteen texture features of DC, AC, and RT images at optimal spatial frequency were extracted based on the GLCM method. Four classification models including PLS-DA, SVM, LS-SVM, and KNN were established using the extracted features. In order to simplify the model, x-loading weights were used to select the effective features, and the corresponding classification models were also constructed using the selected features. Finally, the performance of all models was evaluated to determine the most suitable one for distinguishing between decayed and healthy oranges.

### Classification performance evaluation

Three indexes including *Sensitivity, Specificity*, and *Accuracy* (ACC) were used to evaluate the classification performance of models. For the calibration set or test set, the *Sensitivity* corresponds to the classification accuracy of decayed oranges (true positive rate, TPR), the *Specificity* corresponds to the classification accuracy of healthy oranges (true negative rate, TNR), and ACC represents the classification accuracy of all samples. These evaluation parameters can be calculated as follows:


(4) $$Sensitivity=TPR=\frac{TP}{TP+FN}$$

(5) $$Specificity=TNR=\frac{TN}{TN+FP}$$

(6) $$Accuracy=ACC=\frac{TP+TN}{TP+TN+FP+FN}$$

where *TP* is the number of the decayed oranges correctly classified, *FN* is the number of the decayed oranges incorrectly classified as healthy, *TN* is the number of the healthy oranges correctly classified, and *FP* is the number of the healthy oranges incorrectly classified as diseased.

## Results and discussion

### Demodulated images and analysis

**FIGURE 4:**
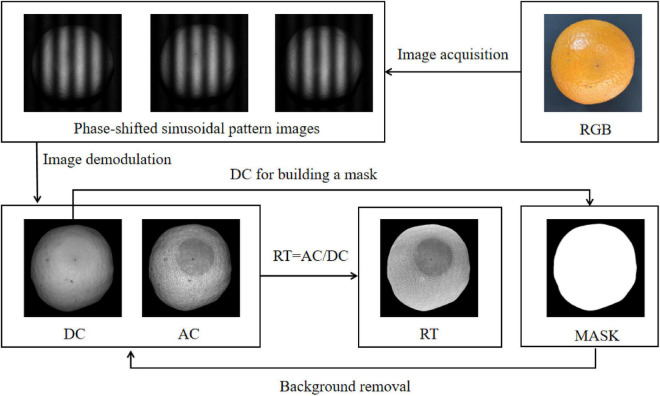
The structured light image processing including image demodulation, background removal, and image ratio.

Figure 4 shows the whole process of image processing. From the original RGB image, it can be seen that the decayed area of orange showed similar color features to the healthy area. Three-phase-shifted structured light images shown in the upper left corner of Figure 4 were acquired from the SIRI system. The phase offsets were − 2π/3, 0, and 2π/3, respectively. By image demodulation, DC and AC images were obtained. It can be observed that the decayed area was almost invisible in the DC image (similar to uniform field illumination), whereas it was obvious in the AC image, indicating that the AC image can be used for effective detection of decay on oranges. In fact, the AC image represents the image containing the specific depth tissue information matched with the used structured light frequency. In this study, although black background was used in the process of image acquisition, it is inevitable that there may be some potential noises. Therefore, background segmentation was performed. The mask background segmentation method was used, and the DC image was used for template creation due to the clear contrast between the orange object and background. It can be noticed from the mask image shown in Figure 4 that only the orange object was completely segmented. Thus, a simple multiplication operation, that is, mask multiplied by the DC (or AC) image, can realize the background removal of the original DC (or AC) image. The RT image is also shown in Figure 4. Compared with DC and AC images, the contrast between the decaying area and the surrounding healthy surface in the RT image was more obvious. More importantly, in the RT image, the surface illumination of the overall orange was relatively uniform, which was conducive to the accurate extraction of texture features and the establishment of effective classification models.

### Selection of spatial frequency

**FIGURE 5:**
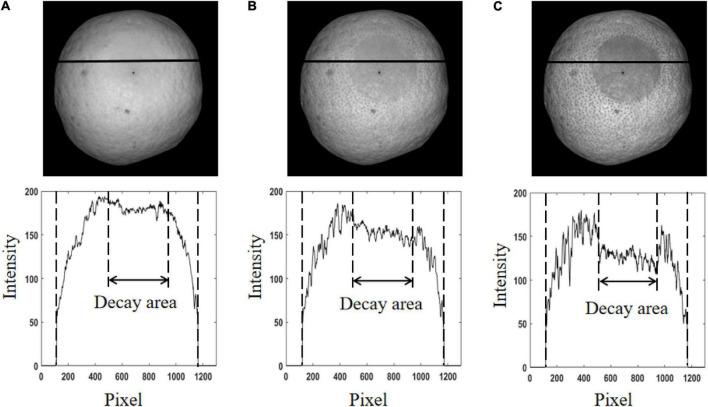
The demodulated AC images and intensity distribution curves. **(A)** The spatial frequency of 0.05 cycles mm^–1^. **(B)** The spatial frequency of 0.15 cycles mm^–1^. **(C)** The spatial frequency of 0.25 cycles mm^–1^.

Figure 5 shows the demodulated AC images and intensity distribution curves. In detail, Figures 5A–C show AC images at three spatial frequencies of 0.05, 0.15, and 0.25 cycle mm^–1^, respectively, and the pixel intensity curves corresponding to the black solid lines in the AC images. The pixel intensity curve was shown at the bottom of each AC image. It corresponded to all pixels on the black solid line across the decayed area in the corresponding AC image. By visually inspecting three AC images, the contrast between the decayed area and healthy area increased gradually with the increase of frequency. It indicated that the accurate recognition of decayed oranges was directly related to the used spatial frequency of structured light. For the three frequencies studied, the frequency of 0.25 cycles mm^–1^ was the best one. However, it can also be seen that the characteristics of the orange epidermis, such as lenticels, were also enhanced with the increase of frequency, which may negatively affect the extraction of texture features. The change of intensity on the sample surface, especially the pixel intensity difference between healthy area and decayed area, can be observed from the pixel intensity distribution as shown in Figure 5. Due to the uneven brightness on the orange surface, the pixel intensity value of the edge region of the sample was usually lower than that of the middle region. From the intensity curve, it can be seen that the pixel intensity of the edge area of the sample in the AC image was even lower than that of the defect area. Therefore, it was difficult to segment defects by using the threshold method. According to three pixel intensity curves, the pixel intensity difference between the decayed area and healthy area of orange in the AC image at 0.25 cycles mm^–1^ was the largest. The reason may be that the decaying tissue was located on the surface or sub-surface of the fruit, and the penetration depth of light into the fruit tissue gradually focused on the orange surface with the increase of frequency, which was helpful to detect the decaying tissue. By comparing the pixel intensity difference between healthy and decayed pixels, the spatial frequency of 0.25 cycles mm^–1^ was determined as the optimal one for subsequent analysis.

### Ratio images and demodulation images of the representative samples

**FIGURE 6:**
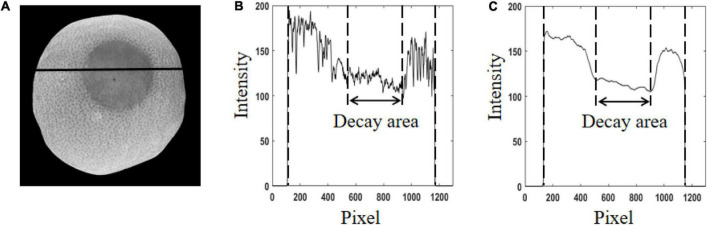
The ratio image and intensity distribution curves. **(A)** The spatial frequency of 0.05 cycles mm^–1^. **(B)** Intensity distribution curves. **(C)** Intensity distribution curves after filtering.

Figure 6 shows the ratio image and intensity distribution curves. The RT image at the spatial frequency of 0.25 cycle mm^–1^ and the pixel intensity curve corresponding to the black solid line in the RT image are shown in Figures 6A,B, respectively. Figure 6C shows the intensity distribution curve of pixels after median filtering with 3 × 3 structural element. Compared with the AC image in Figure 5C, the uneven surface brightness on orange in the AC image was significantly improved in the RT image. In Figure 6B, it can be seen that the low intensity of the edge area was raised to a higher level, and the pixel intensity difference between the healthy and decay areas was more obvious compared with Figure 5C. However, it should also be noted that the noise caused by orange surface lenticels always exists in the RT image. After filtering, this noise was effectively eliminated, as shown in Figure 6C. This result showed that image ratio processing and filtering can significantly improve the uneven illumination on the fruit surface and increase the intensity contrast between the decayed and healthy areas.

**FIGURE 7:**
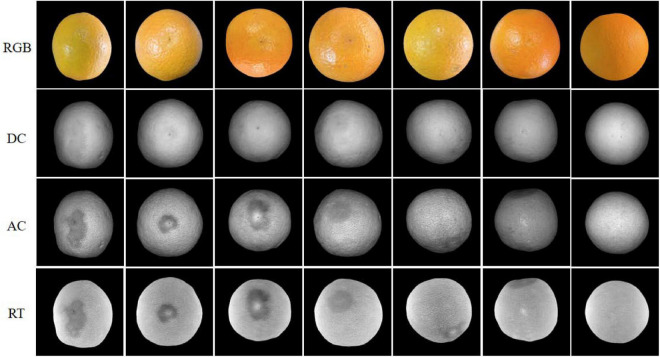
RGB, DC, AC, and RT images of the representative orange samples.

As an example, RGB, DC, AC, and RT images of seven typical samples are shown in Figure 7. The first six were decayed samples and the last one was a healthy sample. In the actual detection, there were differences in the decay degree of oranges, and the decayed areas on oranges were also randomly distributed in the field of view of the camera. Therefore, the selected six decayed samples contained different sizes of decay spots, and these spots were randomly distributed on orange in the image. It can be seen in Figure 7 that it was not easy to identify any decayed region in RGB and DC images, which showed that it was difficult to detect the early decay of oranges by traditional color and monochrome machine vision systems. However, all the decayed areas were clearly seen in AC and RT images. It indicated that the structured light reflection imaging technology can be effectively used to detect decayed oranges, and the detection ability was rarely affected by the degree of decay and location of defect distribution. However, the detection ability for all samples needs to be further verified.

### Classification results based on full features

In order to further ascertain the ability of SIRI technology to detect the early decay of oranges, four classification models including PLS-DA, SVM, LS-SVM, and KNN were established based on fourteen texture features extracted from independent DC, AC, and RT images. All models and the corresponding classification results for samples in the calibration set and the test set are listed in Table 3. For PLS-DA models, the number of the optimal LVs was 13, 9, and 14, respectively, when DC, AC, and RT images were used for modeling. For SVM models, the optimal combination of (C/g) was (9.19/0.099), (1024/0.01), and (891.44/0.01) in terms of three types of input images. For LS-SVM models, the optimal combination of (γ/σ^2^) was (767.98/175.99), (379287.2/1451.86), and (1585313.66/2344.96), respectively. For KNN models, the corresponding *K* value was 5, 1, and 1 for three types of input images, respectively. The classification performance of all models was evaluated according to the indexes of TPR, TNR, and ACC. It can be seen that the results of different classification models were quite different. For texture features extracted from DC and AC images, the performance of the LS-SVM model was the best, and TPR, TNR, and ACC of the model were 81.3, 96.9, and 88.6%, and 89.3, 95.4, and 92.1%, respectively. For texture features extracted from RT images, the PLS-DA model obtained the optimal classification result with TPR, TNR, and ACC of 96.7, 97.7, and 97.1%, respectively. In general, for each type of classifier, the model established based on DC image texture features was the worst, followed by the classification model established based on AC image texture features, and the classification model established based on RT texture features obtained the best classification performance. This result was similar to the analysis in the above sections. Among all the classification models, the PLS-DA model, combined with the RT image, achieved the best classification accuracy. For samples in the prediction set, TPR, TNR, and ACC of the PLS-DA model were 98, 100, and 98.8%, respectively.

**TABLE 3:** Classification results of all oranges based on different models established using full texture features of DC, AC, and RT, respectively.

Input		LVs	(C/g)	(γ/σ^2^)	K	Calibration set					Test set					All samples		
data						Decay (100)	Healthy (100)	TPR (%)	TNR (%)	ACC (%)	Decay (50)	Healthy (30)	TPR (%)	TNR (%)	ACC (%)	TPR (%)	TNR (%)	ACC (%)
DC	PLS-DA	13				85	94	85	94	89.5	34	28	68	93.3	77.5	79.3	93.8	86.1
	SVM		9.19/0.099			86	95	86	95	90.5	30	25	60	83.3	68.8	77.3	92.3	84.3
	LS-SVM			767.98/175.99		89	98	89	98	93.5	33	28	66	93.3	76.3	81.3	96.9	88.6
	KNN				5	86	94	86	94	90	27	25	54	83.3	65	75.3	91.5	82.9
AC	PLS-DA	9				95	95	95	95	95	38	28	76	93.3	82.5	88.7	94.6	91.4
	SVM		1024/0.01			90	94	90	94	92	37	28	74	93.3	81.3	84.7	93.8	88.9
	LS-SVM			379287.2/1451.86		95	97	95	97	96	39	27	78	90	82.5	89.3	95.4	92.1
	KNN				1	88	94	88	94	91	36	28	72	93.3	80	82.7	93.8	87.9
RT	PLS-DA	14				96	97	96	97	96.5	49	30	98	100	98.8	96.7	97.7	97.1
	SVM		891.44/0.01			94	96	94	96	95	44	28	88	93.3	90	92	95.4	93.6
	LS-SVM			1585313.66/2344.96		96	97	96	97	96.5	47	30	94	100	96.3	95.3	97.7	96.4
	KNN				1	91	95	91	95	93	41	27	82	90	85	88	93.8	90.7

### Optimization of features

**FIGURE 8:**
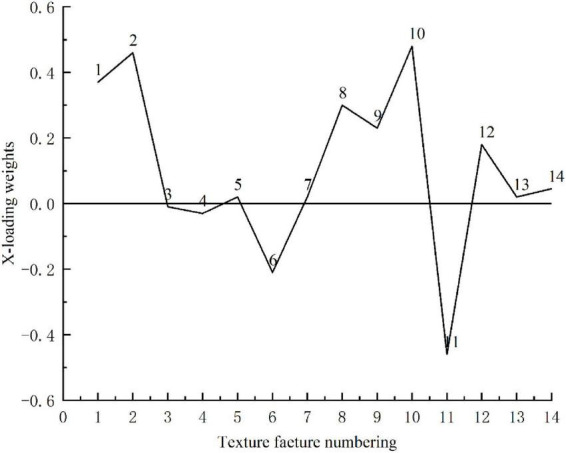
The *x*-loading weights of different features.

The above research showed that an effective model for the classification of decayed oranges can be constructed based on 14 texture features of the RT image, and 14 features were also acceptable for rapid model construction and analysis. However, each texture feature contributed differently to the classification of decayed oranges. Thus, some features may negatively affect the prediction performance (such as accuracy and stability) of the model. Therefore, it was necessary to further optimize the features based on raw 14 texture features. Table 3 indicates the PLS-DA model is the best for the classification of decayed oranges. Thus, the *x*-loading weights of different features extracted from RT images were analyzed to select the effective features. The *x*-loading weight curve is shown in Figure 8. Those texture features, whose absolute value of loading was greater than 0.1 and located at the peak or valley of the loading curve, were selected as important texture features (or called the optimal texture features). According to the above criteria, eight texture features, numbered 1, 2, 6, 8, 9, 10, 11, and 12, were selected. To further evaluate the effectiveness of these features, the selected texture features were used as inputs to establish four kinds of classification models (PLS-DA, SVM, LS-SVM, and KNN), and the results are shown in Table 4. For comparison, the selected eight texture features from DC and AC images were also used as inputs to establish the classification models. The results are also shown in Table 4. By comparing between Tables 3, 4, it can be seen that the classification performance of the models established based on eight features was similar to that of the models established based on fourteen features. Similar to Table 3, the optimal parameters of all models are listed in Table 4. For DC, AC, and RT images, the overall classification accuracy ranges of four types of models were 81.4–85%, 86.4–90.4%, and 92.9–96.4%, respectively. Moreover, similar to the full features models, the PLS-DA model developed based on the eight texture features of the RT image achieved the best classification accuracy. For samples in the prediction set, TPR, TNR, and ACC of the PLS-DA model were 98, 100, and 98.8%, respectively. These results were the same as the full-feature PLS-DA model, which further showed that feature selection can optimize the classification model.

**TABLE 4:** Classification results of all oranges based on different models established using the selected eight important texture features of DC, AC, and RT, respectively.

Input		LVs	(C/g)	(γ/σ^2^)	K	Calibration set					Test set					All samples		
data						Decay (100)	Healthy (100)	TPR (%)	TNR (%)	ACC (%)	Decay (50)	Healthy (30)	TPR (%)	TNR (%)	ACC (%)	TPR (%)	TNR (%)	ACC (%)
DC	PLS-DA	8				83	93	83	93	88	31	26	62	86.7	71.3	76	91.5	83.2
	SVM		1024/0.16			83	92	83	92	87.5	28	25	56	83.3	66.3	74	90	81.4
	LS-SVM			256709.5/94.99		90	91	90	91	90.5	33	24	66	80	71.3	82	88.5	85
	KNN				5	79	100	79	100	89.5	22	27	44	90	61.6	67.3	97.7	81.4
AC	PLS-DA	8				89	91	89	91	90	40	24	80	80	80	86	88.5	87.1
	SVM		1024/0.07			93	95	93	95	94	38	27	76	90	81.3	87.3	93.8	90.4
	LS-SVM			5925.38/672.96		93	94	94	94	93.5	38	26	76	86.7	80	87.3	92.3	89.6
	KNN				1	88	92	88	92	90	38	24	76	80	77.5	84	92.2	86.4
RT	PLS-DA	8				94	97	94	97	95.5	49	30	98	100	98.8	95.3	97.7	96.4
	SVM		776.05/0.01			95	96	95	96	95.5	45	28	90	93.3	91.3	93.3	95.4	94.2
	LS-SVM			2451928.8/3169.78		95	97	95	97	96	46	30	92	100	95	94	97.7	95.6
	KNN				7	92	96	92	96	94	44	28	88	93.3	90	90.7	95.4	92.9

In previous studies on early decay detection in citrus, hyperspectral imaging was one of the most attractive technologies and achieved superior detection performance. For example, Li et al. (2019) used visible and near-infrared hyperspectral imaging to identify the early decayed oranges. Seven wavelength images in the spectral region of 500–1,050 nm were finally determined to develop the multispectral image detection algorithm and obtained classification accuracies of 97.3 and 100% for decayed and healthy oranges, respectively. Zhang et al. (2020) used a similar system to detect decayed mandarins. Although only two wavelength images were extracted and used in the algorithm, the recognition accuracy of decayed fruit was low (only 90.57%). Luo et al. (2022) proposed a multispectral image processing algorithm for the decayed orange detection combined with four feature wavelength images based on hyperspectral imaging technique, achieving an overall classification accuracy of 98.6%. Although hyperspectral imaging can be used for the detection of decayed citrus fruits, the expensive construction cost and the time-consuming data acquisition/processing greatly limit the practical application of this technology. Wavelength selection is helpful to build a rapid multispectral detection system, but there is no practical application case for multispectral imaging detection of decayed citrus. In fact, there are still many problems to be solved in hyperspectral imaging technology (Lu et al., 2017b). In this study, the constructed SIRI system has low cost and fast data acquisition speed, and this study has indicated that SIRI can obtain similar or better detection performance for the early decay detection in oranges compared with hyperspectral imaging.

## Conclusion

This study proposed a new SIRI technology combined with a visible LED lamp and a monochrome camera and successfully demonstrated the feasibility of detecting oranges with early decay. The spatial frequency of 0.25 cycles mm^–1^ was proved to be superior to spatial frequencies of 0.05^1^ and 0.15 cycles mm^–1^ for detection of the decayed oranges. Compared with DC and AC images, the RT image was more suitable for decay detection due to uniform orange surface illumination and clear contrast between decayed and healthy areas. By using x-loading weight analysis, eight important texture features, namely angular second moment, contrast, sum average, sum entropy, entropy, difference variance, difference entropy, and information measures of correlation, were extracted from 14 texture features. Four types of classification models, including PLS-DA, SVM, LS-SVM, and KNN, were established using all the fourteen texture features and the selected eight texture features based on DC, AC, and RT images. The classification results indicated that four kinds of models based on the RT image had better performance than those models established based on DC and AC images. Therefore, it can be considered that the RT image can improve the ability of the original DC or AC image in the detection of decayed oranges. Among all models, the PLS-DA model, combined with the RT image, achieved the best classification accuracy, regardless of the models established based on full features or important features. For all samples, the PLS-DA model with full features and the selected eight features obtained classification accuracy of 97.1 and 96.4%, respectively. The similar classification accuracy indicated that the selected eight texture features could be used to construct the models for the detection of oranges with early decay. All results confirmed that the proposed SIRI technology based on a visible LED lamp and a monochrome camera, combined with texture feature classification models, can be used to identify oranges with early decay.

## Data availability statement

The original contributions presented in this study are included in the article, further inquiries can be directed to the corresponding author.

## Author contributions

ZC: methodology, system development, and original manuscript writing. WH: experimental equipment and funding. QW: system development. JL: methodology, editing, revision, supervision, and funding. All authors contributed to the article and approved the submitted version.
